# Supplementary material for: Wheat chromatin architecture is organized in genome territories and transcription factories
Source: Genome Biol. 2020 Apr 29;21:104. doi: 10.1186/s13059-020-01998-1 (PMC7189446; doi:10.1186/s13059-020-01998-1)
Supplement: Supplementary file 1 — Additional file 1. Supplementary figures. [file 13059_2020_1998_MOESM1_ESM.pdf]

Supplemental Figure S1

Shoot Hi-C

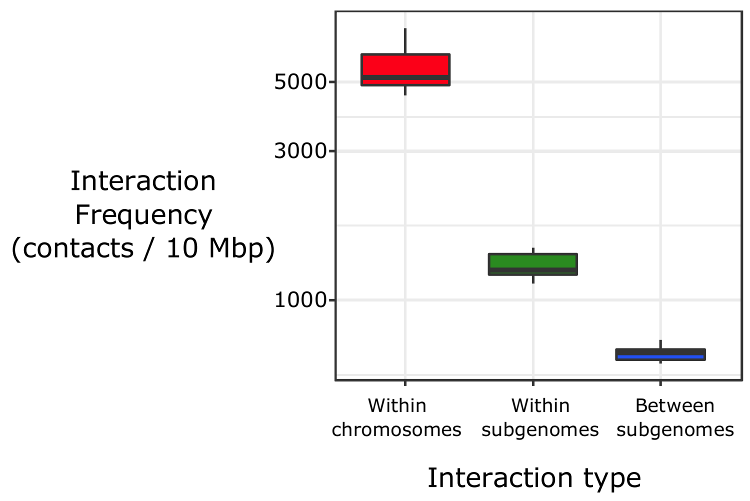

Root Hi-C

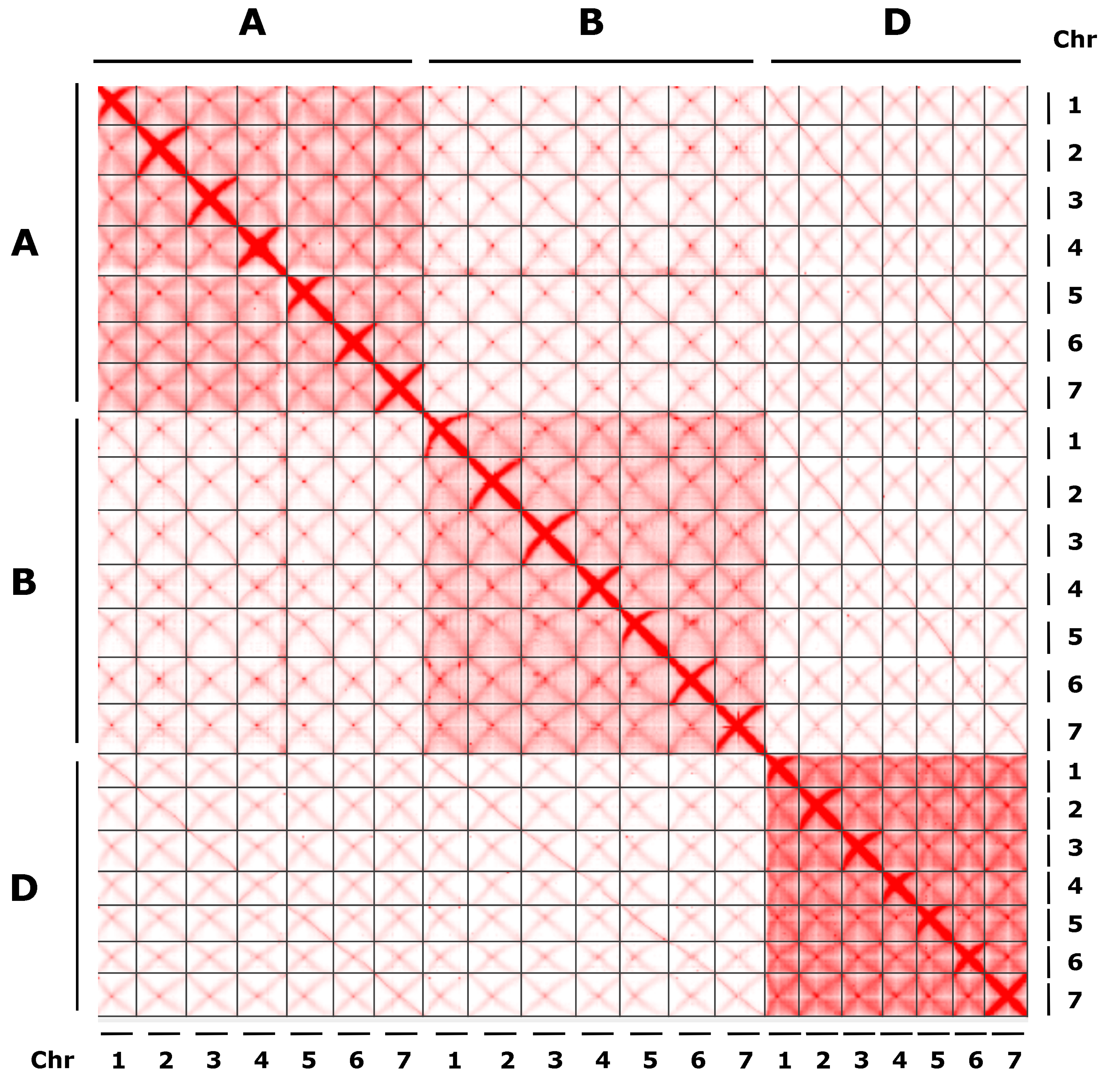

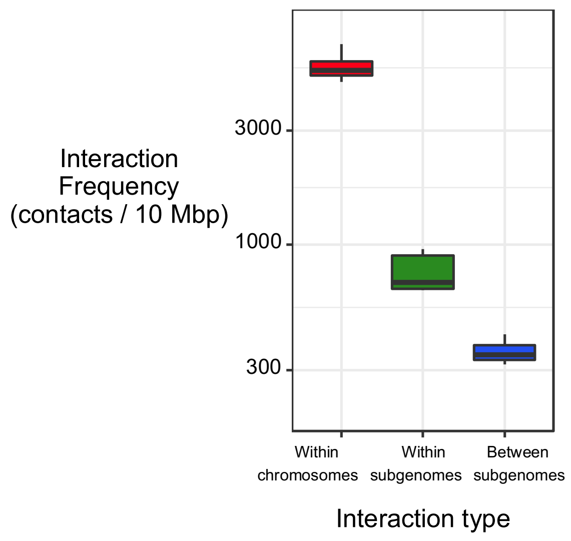

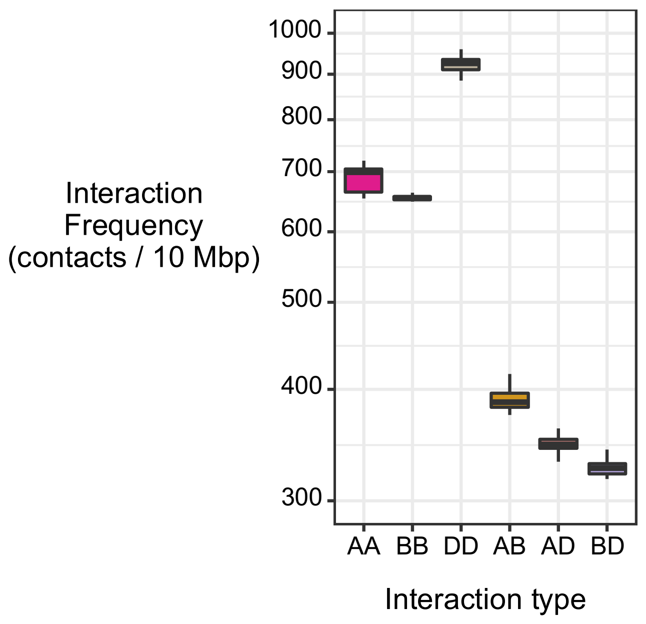

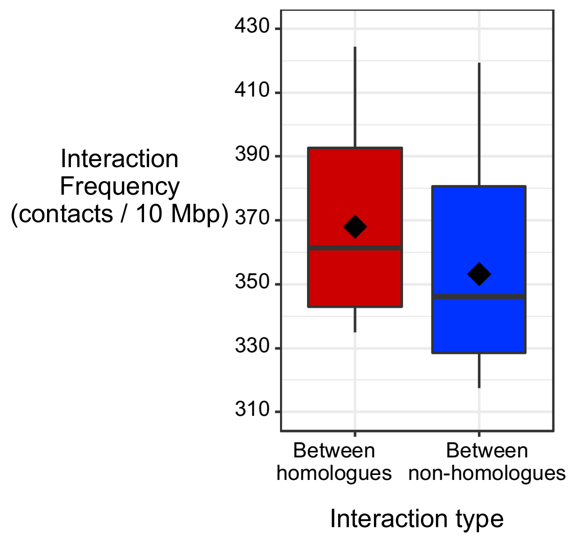

Supplemental Figure S2

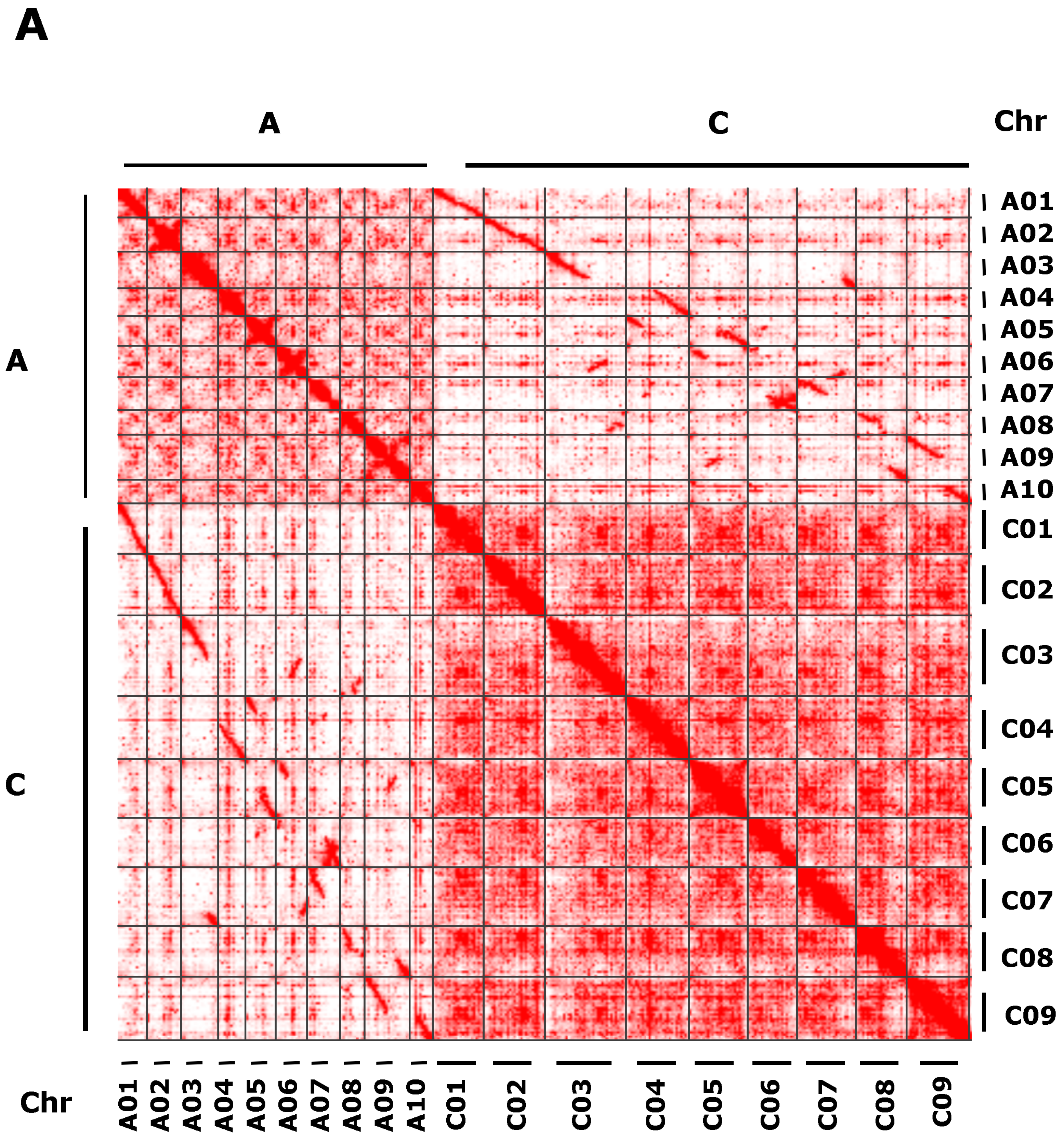

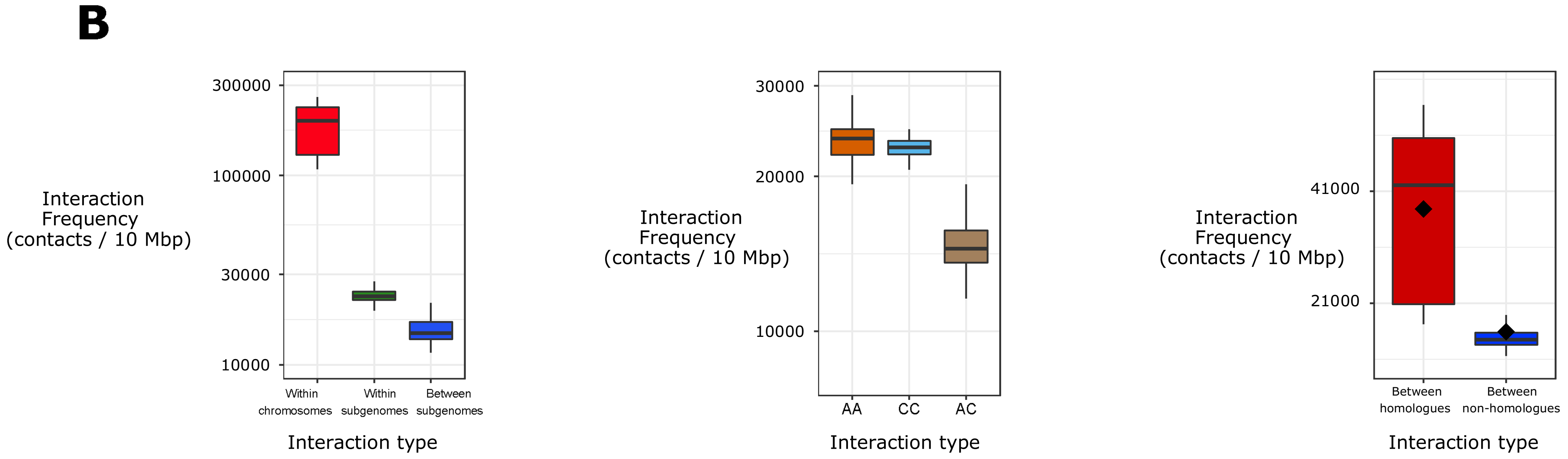

Supplemental Figure S3

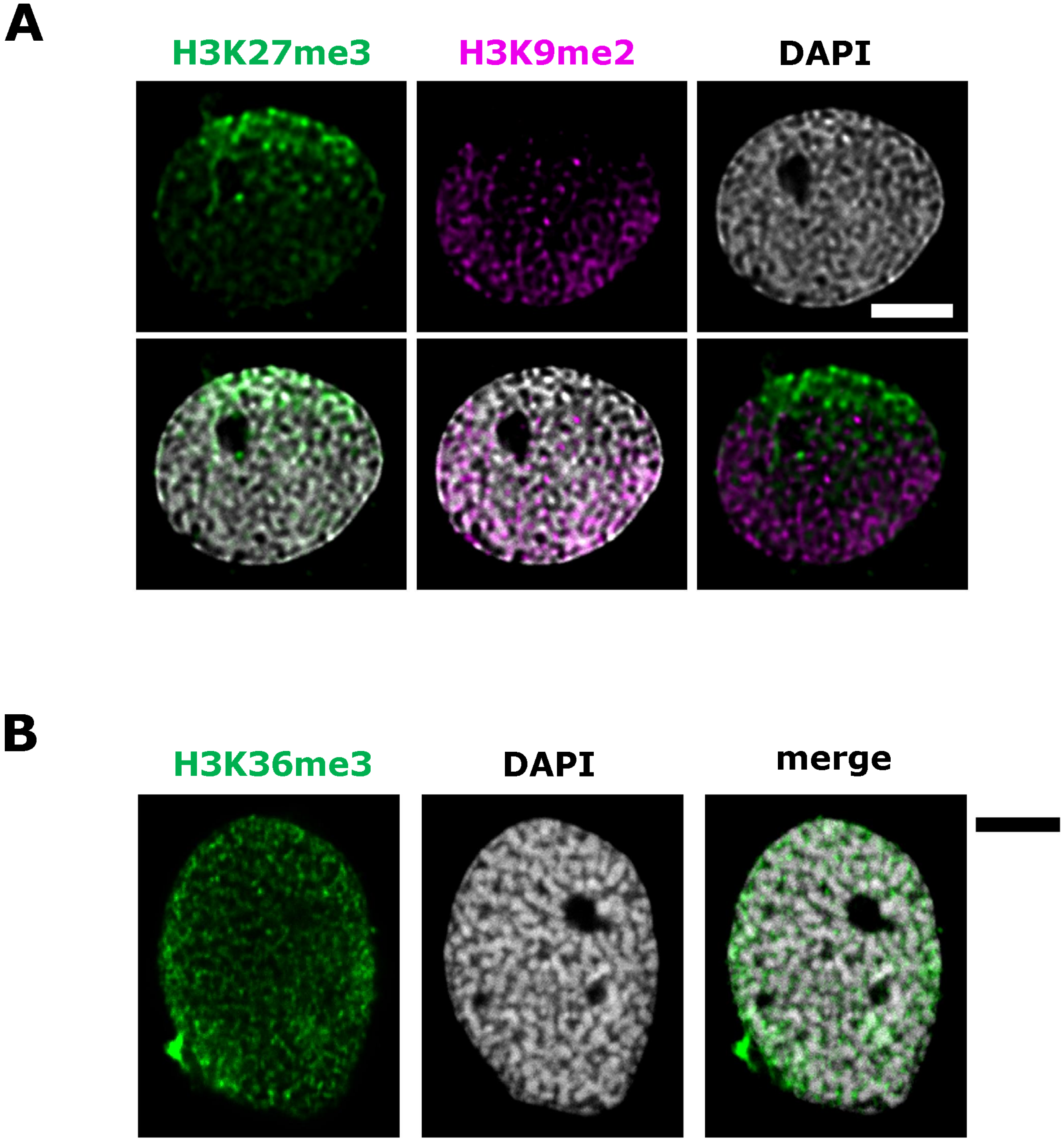

Supplemental Figure S4

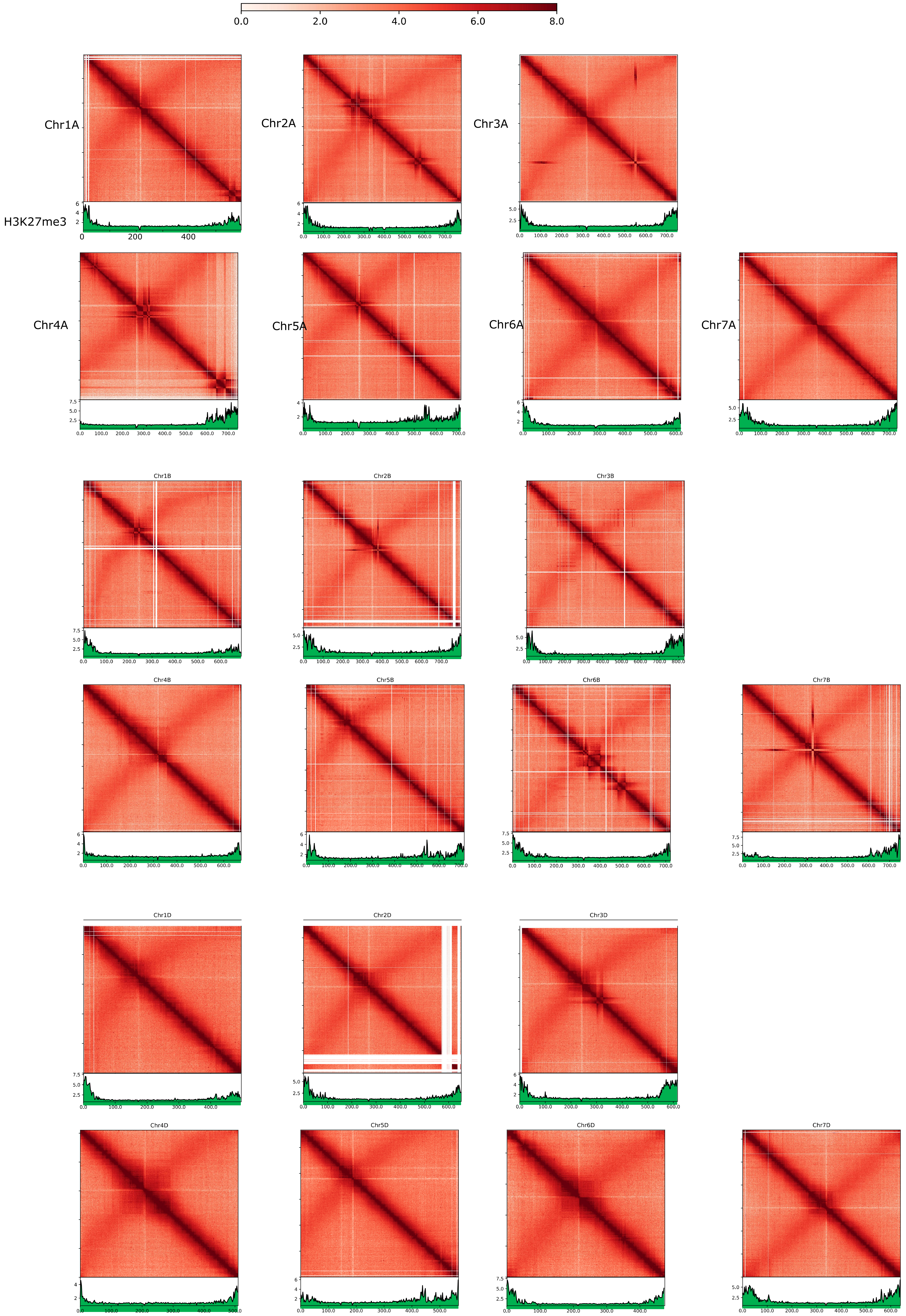

Supplemental Figure S5

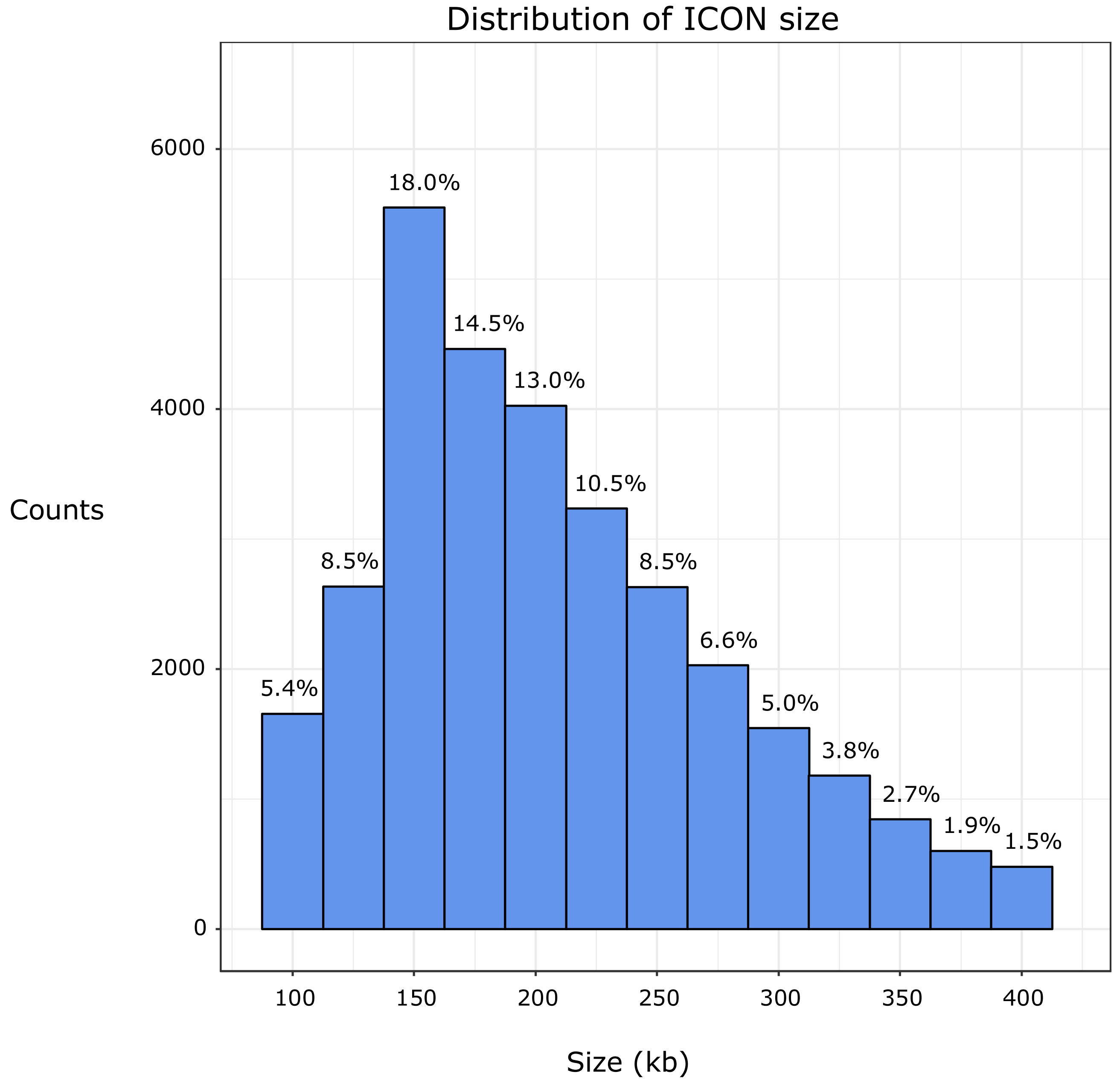

Supplemental Figure S6

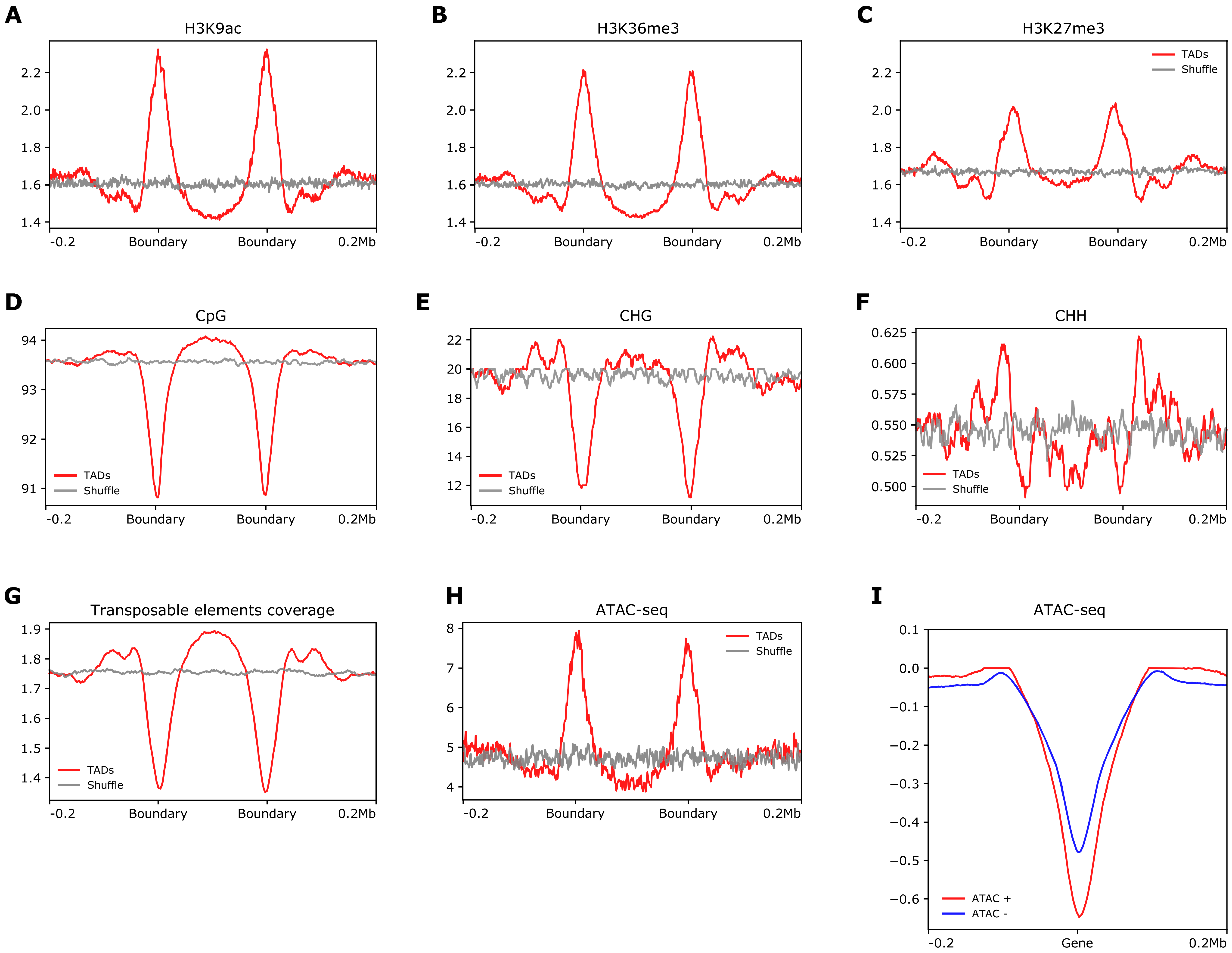

Supplemental Figure S7

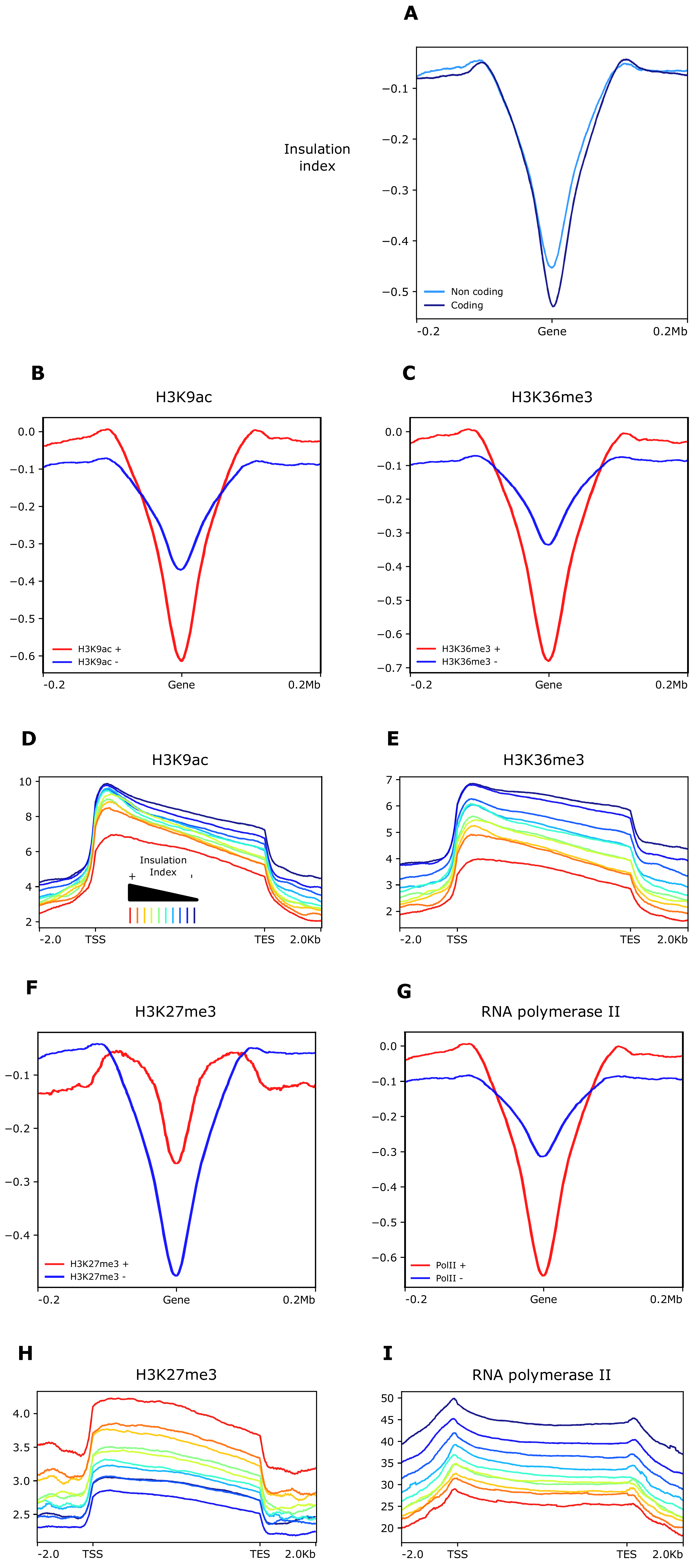

Supplemental Figure S8

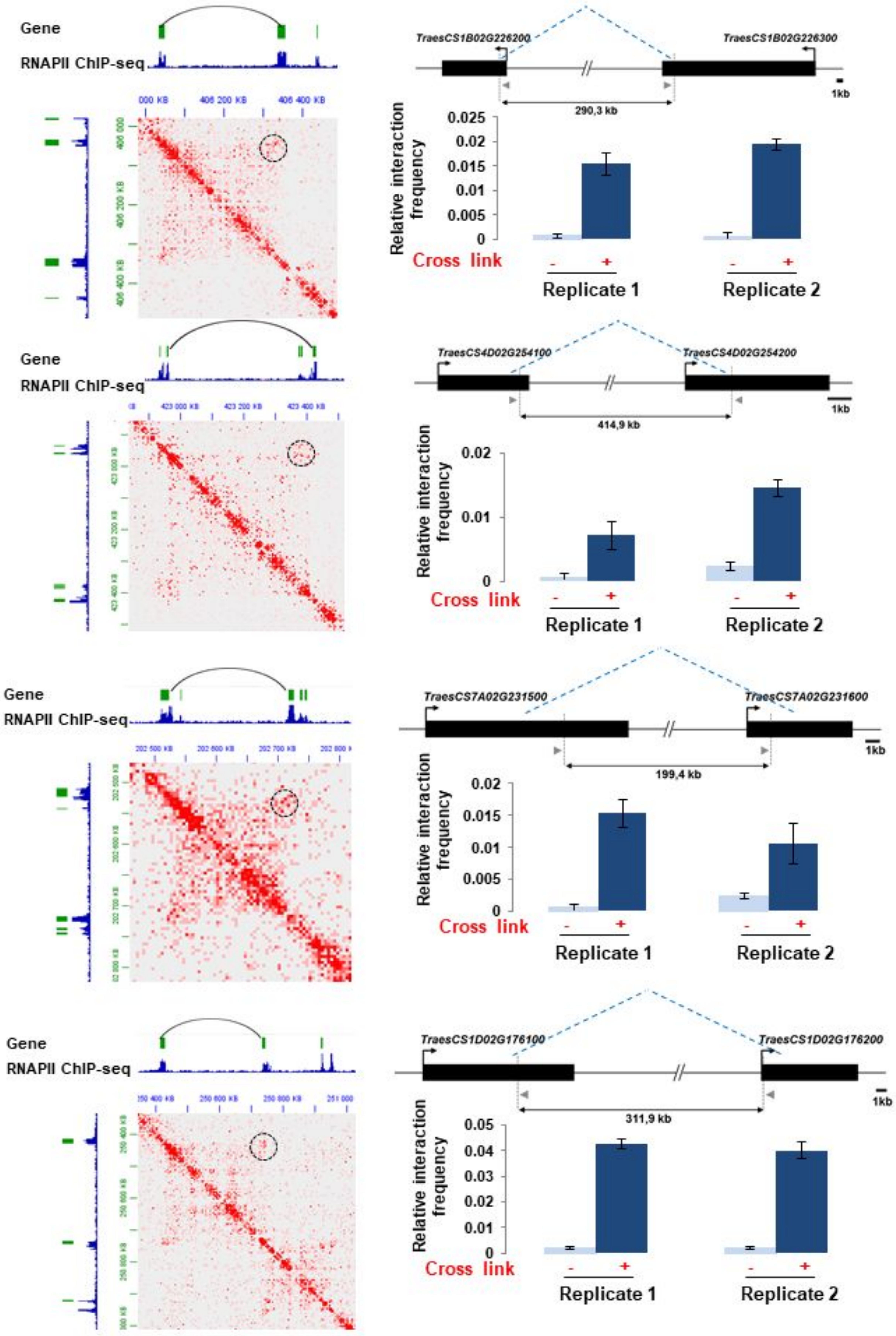

Supplemental Figure S9

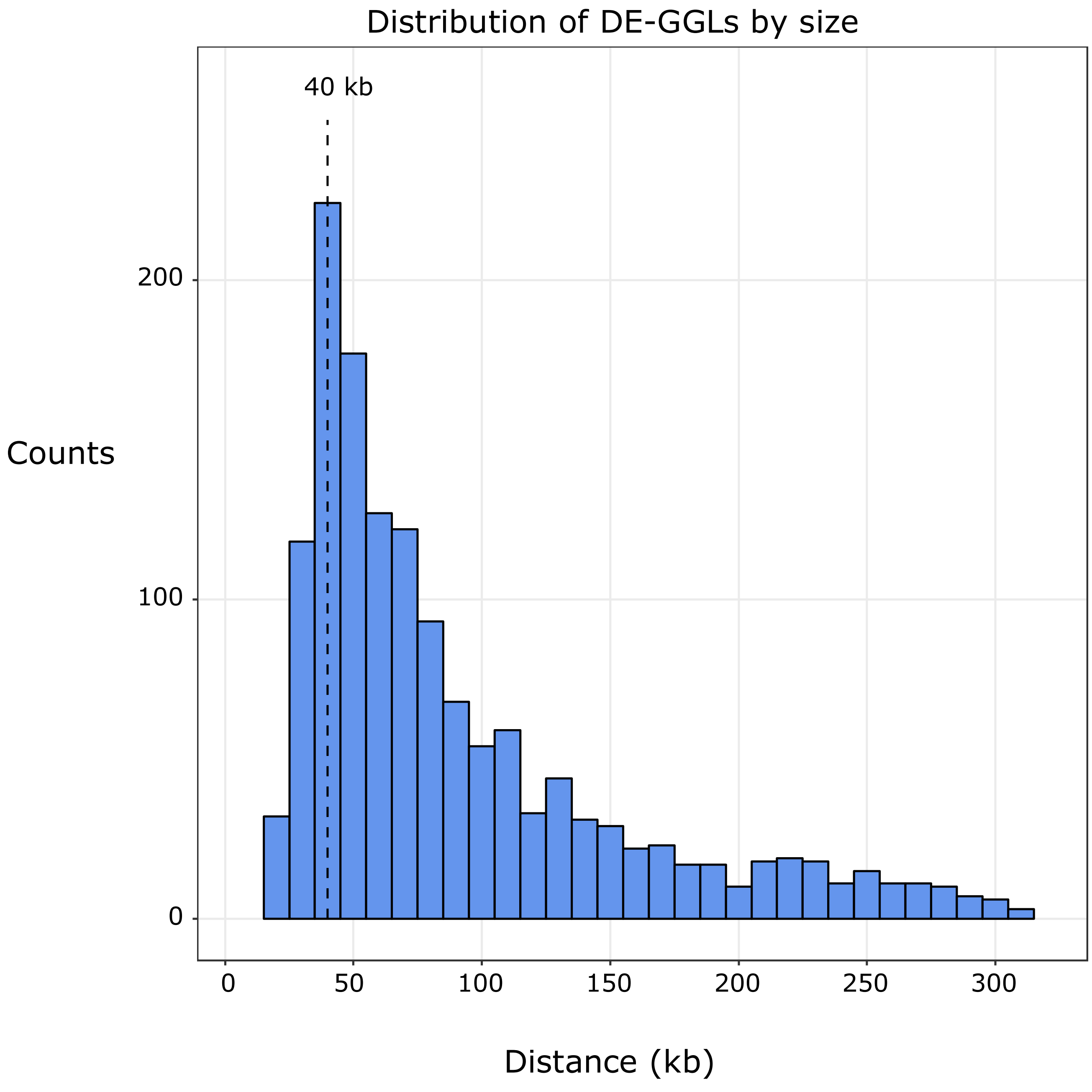

Supplemental Figure S10

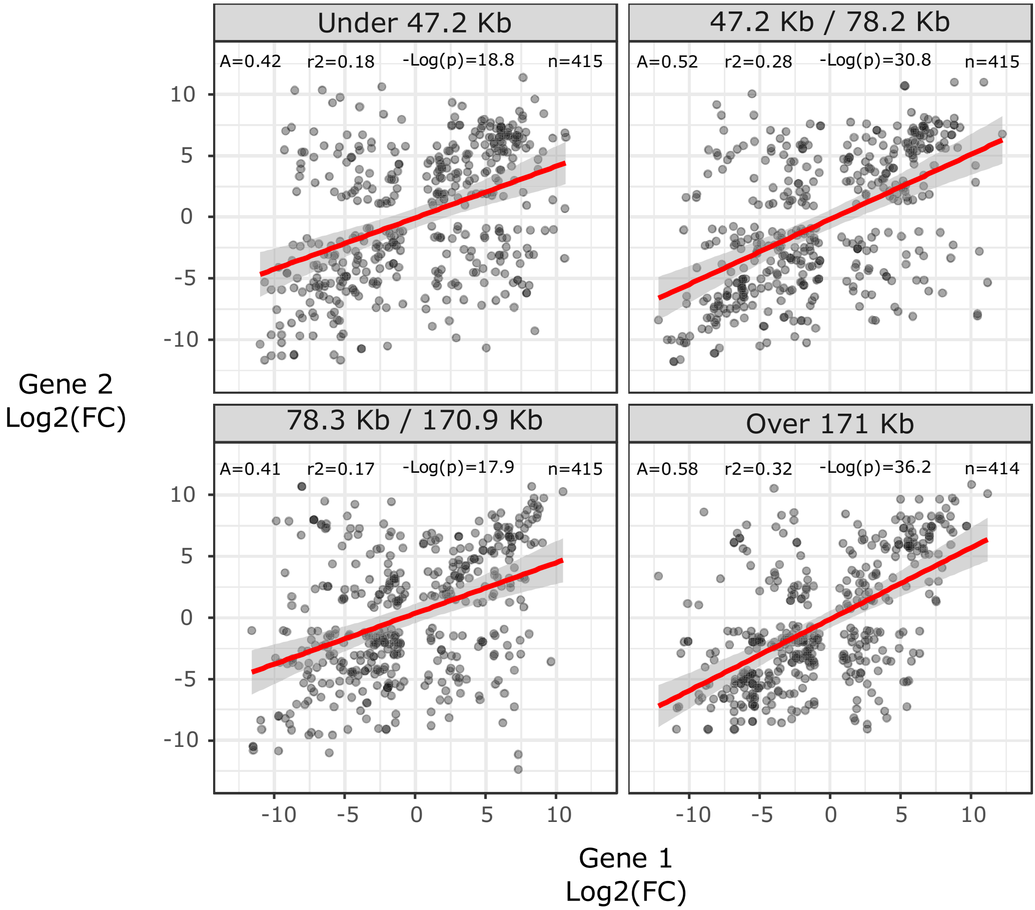

Supplemental Figure S11

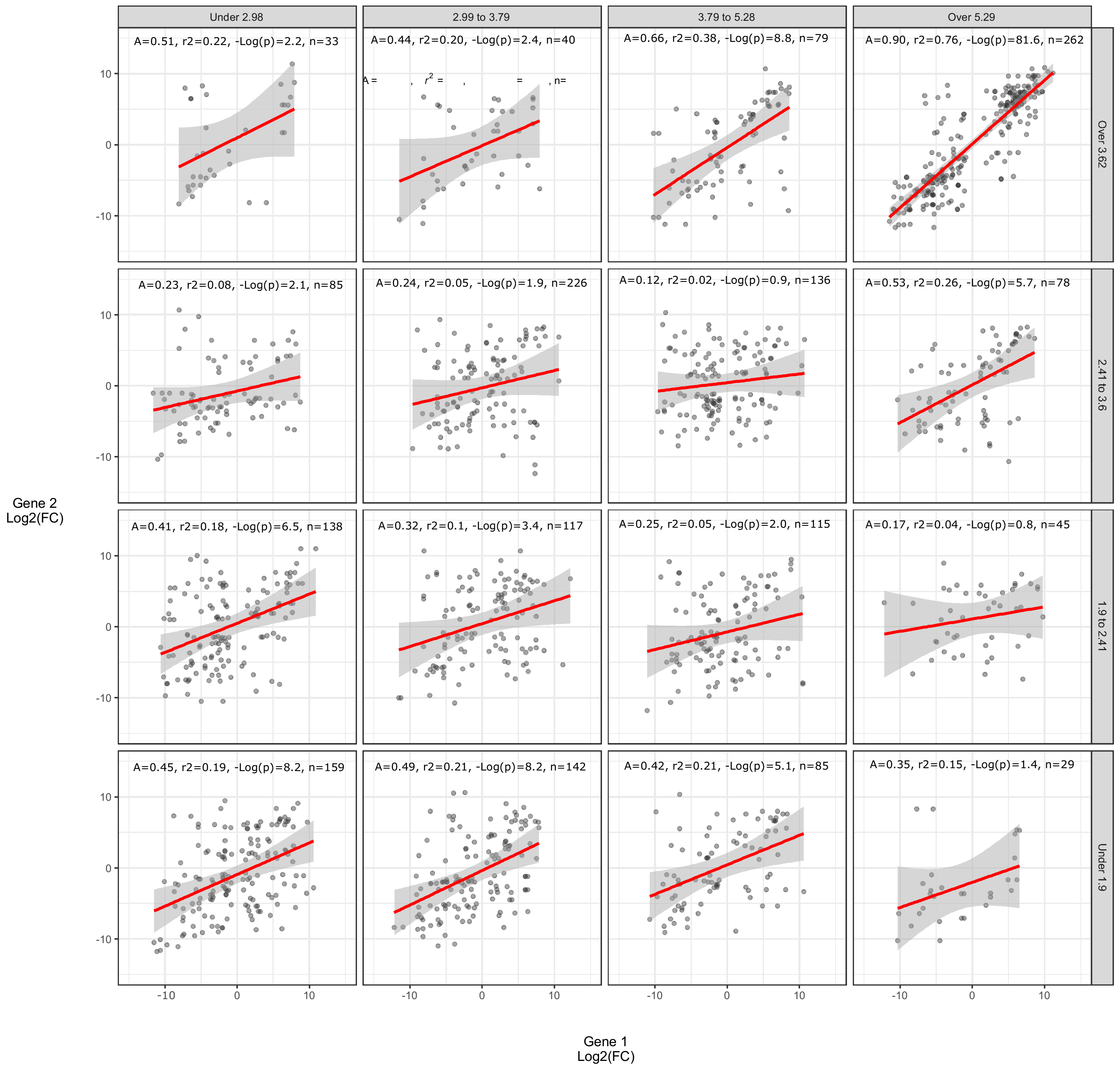

Supplemental Figure S12

A

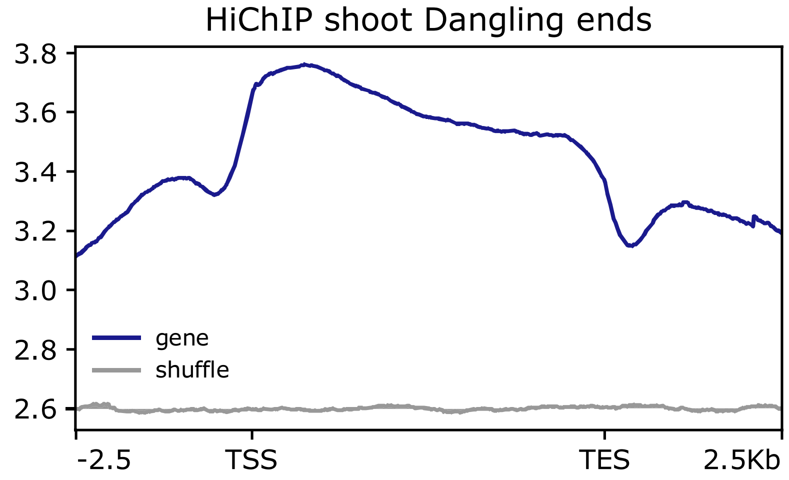

B

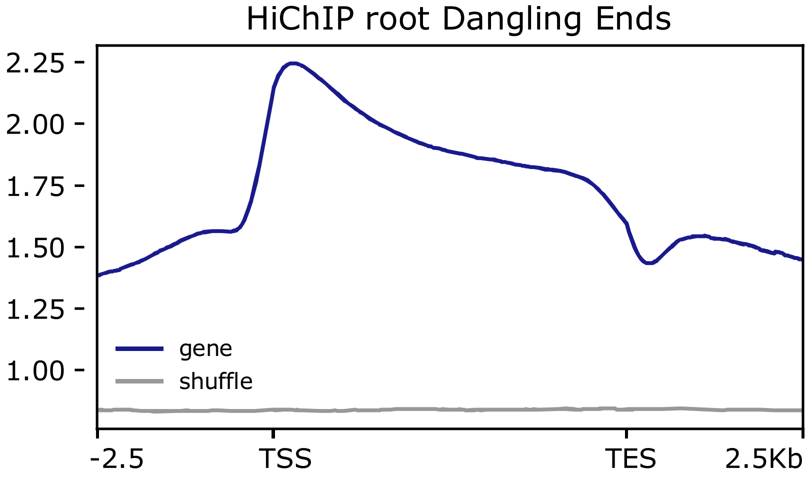

C

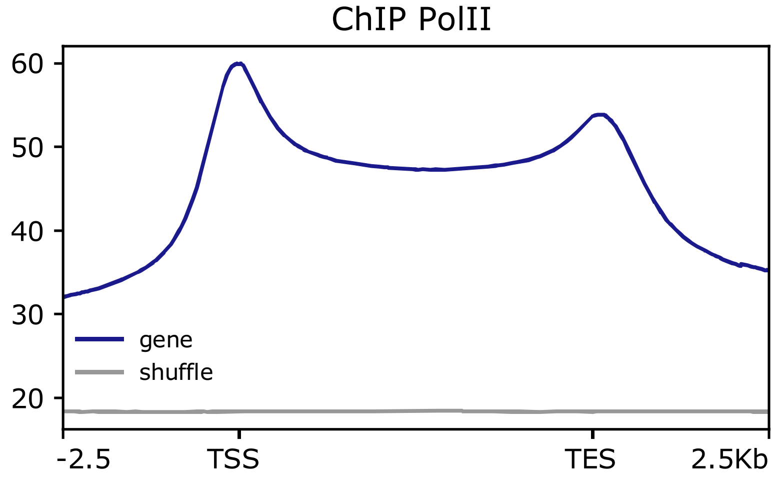

Supplemental Figure S13

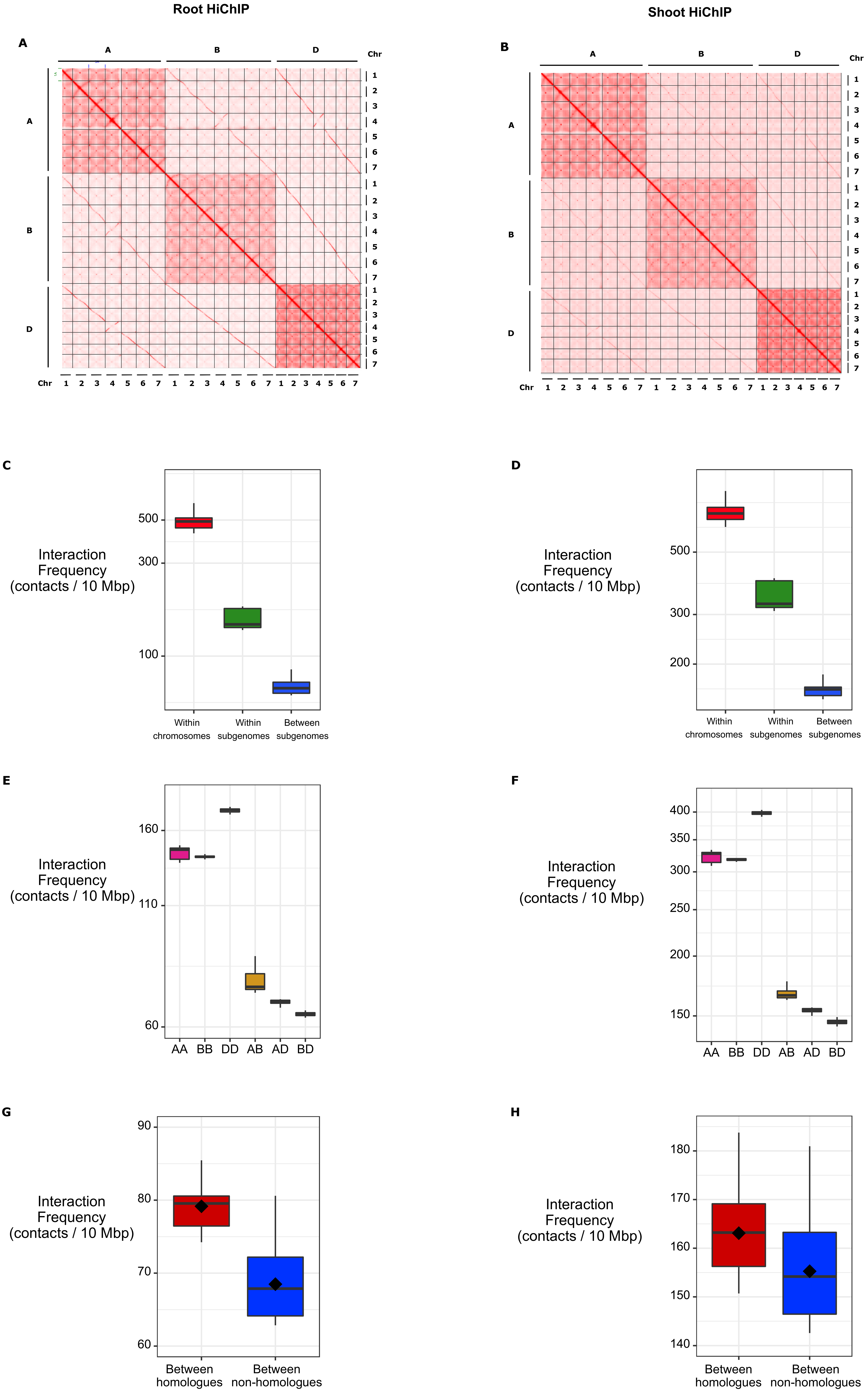

Supplemental Figure S14

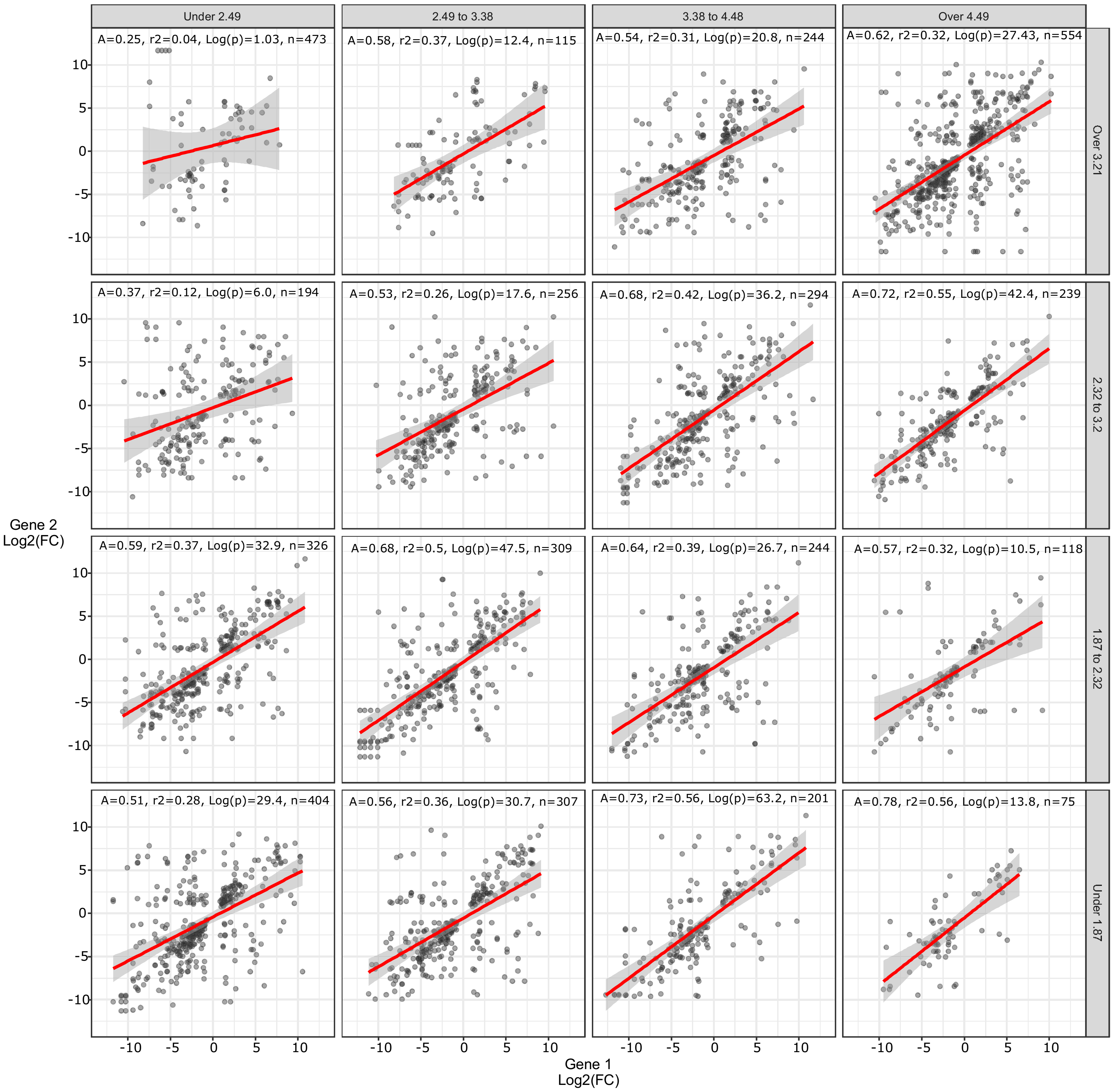

SUPPLEMENTAL FIGURES LEGENDS

Supplemental Figure S1. Large-scale chromatin architecture analysis of hexaploid wheat shoot and root

A) Box plots representing the distribution of the median interaction frequency for intrachromosomal interactions, interchromosomal interactions within each subgenome, and interchromosomal interactions between subgenomes in shoot nuclei. B) Whole- genome interaction matrix obtained from root nuclei. C) Box plots showing the distribution of the median interaction frequency in root nuclei for different groups of interactions: intrachromosomal, interchromosomal within each subgenome, and interchromosomal between subgenomes (left); between different combinations of subgenomes (center); between homoeologous and nonhomoeologous chromosomes of different subgenomes (right).

Supplemental Figure S2. Large-scale chromatin architecture analysis of rapeseed (Brassica napus L.)

Whole-genome Hi-C interaction matrix obtained from rapeseed nuclei (upper panel). Box plots showing the distribution of the median interaction frequency in rapeseed nuclei for different groups of interactions (bottom panel): intrachromosomal, interchromosomal within each subgenome, and interchromosomal between subgenomes (left); between different combinations of subgenomes (center); between homoeologous and nonhomoeologous chromosomes of different subgenomes (right).

Supplemental Figure S3. Distribution of chromatin marks within the nucleus of wheat.

A) Immunofluorescence detection of H3K27me3 (green) and H3K9me2 (purple) and DAPI (white) in isolated nucleus. Scale bar represents 10 pm. B) Immunofluorescence detection of H3K36me3 (green) and DAPI (white) in isolated nucleus. Scale bar represents 10 pm.

Supplemental Figure S4. Single-chromosome Hi-C contact matrixes

Hi-C contact matrixes for all chromosomes of wheat along with the short read density of H3K27me3 ChIP-Seq (in green). The pattern of strong diagonal and anti-diagonal signal is visible, together with the H3K27me3 enrichment in the subtelomeric regions.

Supplemental Figure S5. Distribution of ICONS by size.

Histograms showing the number and percentage of ICONS for different classes of size.

Supplemental Figure S6. Relation between ICONs, epigenetic and genomic features. Metaplots showing the normalized short-read density of H3K9ac (A), H3K36me3 (B), H3K27me3 ChIP-Seq (C), CpG (D), CHG (E), and CHH methylation percentage (F), TE coverage (G), and ATAC-Seq shortread density on ICONs (red) and a random subset of sequences (grey). I) Metaplots showing the median insulation index on genes with (red) or without (blue) a peak of ATAC-Seq read density within 500base pairs upstream of the TSS.

Supplemental Figure S7. Relation between insulation index and non-coding genes (ncRNA)

(A) Metaplots showing the median insulation index on protein-coding genes (PCGs) (dark blue) and non-coding genes (ncRNAs) (light blue). (B-C) Metaplots showing the median insulation index on non-coding genes overlapping (red) or nonoverlapping (blue) with a peak of H3K9ac and H3K36me3 ChIP-Seq, respectively. (D-E) Metaplots showing normalized ChIP-Seq short-read density of H3K9ac and H3K36me3, respectively, on non-coding genes with increasing insulation index (see Materials and Methods). (F-G) Metaplots showing the median insulation index on non-coding genes overlapping (red) or nonoverlapping (blue) with a peak of H3K27me3 and RNA polymerase II ChIP-Seq, respectively. (H-I) Metaplots showing normalized ChIP-Seq short-read density of H3K27me3 and RNA polymerase II, respectively, on non-coding genes with increasing insulation index (see Materials and Methods).

Supplemental Figure S8. Validation of gene to gene loop interaction by chromosome conformation capture assay followed by quantitative PCR (3C-QPCR).

Right: 2D heatmap with the frequency of intrachromosomal interactions are presented for four gene to gene loop interactions namely (TraesCS1B02G226200 - TraesCS1B02G226300 ; TraesCS4D02G254100 - TraesCS4D02G254200 ; TraesCS7A02G231500 - TraesCS7A02G231600 ; TraesCS1D02G176100 - TraesCS1D02G176200. Circular arcs highlight the interaction find by Hi-C analysis. Green bars represent genes. The dashed blue circle highlights the interaction in the 2D heat map.

Left: Schematic representation of the interacting loci. The position of each primer pair used for 3C-qPCR is indicated by a grey arrow. The black arrow indicates the position of the transcription start site. The dash grey line indicates the position of the DpnII restriction site for each locus. The dashed blue line pointed the tested interaction. Relative interaction frequencies were calculated as described in Materials and Methods. Two biologicals replicates are presented.

Supplemental Figure S9. Distribution by size of gene-to-gene loops between differentially expressed genes.

Supplemental Figure S10. Effect of gene-to-gene loop size on coregulation of differentially expressed genes.

Scatterplots between log2 (fold-change shoot/root) of differentially expressed gene pairs associated to gene-to-gene loops of different size. GGLs between DE genes were partitioned in four quartiles depending on their size and linear regression calculated for each group. Slope, R^2 and statistical significance [log10(p-value)] are similar for all the classes of distance.

Supplemental Figure S11. Effect of gene-to-gene loop strength on coregulation of differentially expressed genes.

Scatterplots between log2 (fold-change shoot/root) of differentially expressed gene pairs associated to gene-to-gene loops of different strength, as calculated by HOMER using expected and observed reads. GGLs between DE genes were partitioned in four quartiles of strength in shoot and, within these, in four quartiles of strength in root and linear regression was calculated for each group. According to the slope and r2, the association is particularly strict for the strongest GGLS in shoot (Z-score >5.29) and root (Z-score > 3.62).

Supplemental Figure S12. HiChIP with RNA polymerase II antibody produces a signal similar to RNA Polymerase II ChIP.

Metaplots showing the short read density on genes (red) and a random subset of sequences (grey) of "dangling ends" from RNAPII HiChiP carried out in shoot nuclei (A), root nuclei (B) and RNAPII ChIP (C). All the experiment produced an enrichment over genes with a local maximum around the TSS.

Supplemental Figure S13. HiChIP with RNA polymerase II antibody confirms the large-scale chromatin architecture described by Hi-C.

(A-B) Whole-genome interaction matrixes obtained with RNAPII HiChIP on nuclei of wheat shoot and root, respectively. (C-D) box plots representing the distribution in shoot and root nuclei, respectively, of the median interaction frequency for intrachromosomal interactions, interchromosomal interactions within each subgenome, and interchromosomal interactions between subgenomes.

(E-F) distribution in shoot and root nuclei of the median interaction frequency between different combinations of subgenomes. (G-H) distribution in shoot and root nuclei of the median interaction frequency between homoeologous and nonhomoeologous chromosomes of different subgenomes.

Supplemental Figure S14. Effect of the strength of interchromosomal RNAPII-associated loops (RALs) on coregulation of differentially expressed genes.

Scatterplots between log2 (fold-change shoot/root) of differentially expressed gene pairs associated to RNAPII-associated loops of different strength, as calculated by HOMER using expected and observed reads (see Materials and Methods). RALs between DE genes were partitioned in four quartiles of strength in shoot and, within these, in four quartiles of strength in root and linear regression was calculated for each group. According to the slope and R^2, the strength of interchromosomal RALs does not affect the coregulation.
